# VE-Cadherin Disassembly and Cell Contractility in the Endothelium are Necessary for Barrier Disruption Induced by Tumor Cells

**DOI:** 10.1038/srep45835

**Published:** 2017-04-10

**Authors:** Virginia Aragon-Sanabria, Steven E. Pohler, Vikram J. Eswar, Matthew Bierowski, Esther W. Gomez, Cheng Dong

**Affiliations:** 1 Department of Biomedical Engineering, Pennsylvania State university, University Park, PA, 16802, USA; 2 Department of Chemical Engineering, Pennsylvania State University, University Park, PA, 16802, USA.

## Abstract

During metastasis, breakdown of the endothelial barrier is critical for tumor cell extravasation through blood vessel walls and is mediated by a combination of tumor secreted soluble factors and receptor-ligand interactions. However, a complete mechanism governing tumor cell transendothelial migration remains unclear. Here, we investigate the roles of tumor-associated signals in regulating endothelial cell contractility and adherens junction disassembly leading to endothelial barrier breakdown. We show that Src mediates VE-cadherin disassembly in response to metastatic melanoma cells. Through the use of pharmacological inhibitors of cytoskeletal contractility we find that endothelial cell contractility is responsive to interactions with metastatic cancer cells and that reducing endothelial cell contractility abrogates migration of melanoma cells across endothelial monolayers. Furthermore, we find that a combination of tumor secreted soluble factors and receptor-ligand interactions mediate activation of Src within endothelial cells that is necessary for phosphorylation of VE-cadherin and for breakdown of the endothelial barrier. Together, these results provide insight into how tumor cell signals act in concert to modulate cytoskeletal contractility and adherens junctions disassembly during extravasation and may aid in identification of therapeutic targets to block metastasis.

Vascular endothelial cells form a physical and dynamic barrier that lines the interior of blood vessels throughout the body and regulates passage of cells and molecules between the blood stream and the surrounding tissues^1^. Elevated permeability of blood vessels is a hallmark of inflammation and of a variety of vascular pathologies including edema, tumor angiogenesis, and sepsis. Multiple studies have shown that metastatic cancer cells are capable of disrupting the endothelium^2^,^3^,^4^. During metastasis, cancer cells must cross the endothelial barrier twice; first during intravasation to get from the primary tumor into the blood stream and second, during extravasation to get from the blood stream into the surrounding tissue to form a secondary tumor at a distant site^5^,^6^,^7^. However, a complete mechanism describing how tumor cells impact endothelial barrier during intravasation and extravasation remains unclear.

Vascular endothelial (VE)-cadherins are the main proteins sustaining intercellular adherens junctions in the vascular endothelium and they modulate endothelial permeability^8^,^9^,^10^. VE-cadherin contains five extracellular domains that form Ca^2+^-dependent homodimer interactions across cell membranes, one transmembrane domain, and a cytoplasmic tail that binds to multiple catenins thereby providing a physical link to the cytoskeleton and enabling mechanotransduction inside the cell^11^,^12^,^13^,^14^. Under certain physiological and pathological conditions, proteins in the cadherin/catenin complex are phosphorylated, which results in dissociation of the complex and ultimately impacts the stability of endothelial cell-cell junctions^12^,^15^,^16^,^17^,^18^,^19^.

During leukocyte transendothelial migration (TEM), VE-cadherins are initially maintained in a de-phosphorylated state supporting adherens junctions; however, around sites of leukocyte TEM, VE-cadherins are phosphorylated and temporarily leave the site of transmigration. These steps have been well characterized as part of the main events leading to endothelial barrier breakdown^20^,^21^,^22^,^23^,^24^,^25^. Interestingly, in the context of cancer metastasis there have been mixed results regarding VE-cadherin phosphorylation and its implications. Using an *in vitro* system, Peng *et al*. showed that metastatic melanoma cells in direct contact with endothelial monolayers failed to induce VE-cadherin phosphorylation following 45 minutes of interaction between cancer cells and endothelial cells^26^. In contrast, Haidari *et al*. reported that invasive breast cancer cells promote phosphorylation of VE-cadherin after only seven minutes^27^. In another study, Adam *et al*. showed that tyrosine phosphorylation of VE-cadherin is not sufficient to decrease barrier function of endothelial monolayers^28^. These seemingly conflicting results may be caused by the different metastatic potentials of the cancer cell lines studied in each case. As such, it is not clear whether cancer cells of different metastatic potentials differentially regulate VE-cadherin phosphorylation thereby disrupting the endothelium to varying degrees.

Endothelial cell-cell junctions are thought to be regulated by a balance between cell-cell adhesion and cell contractility^29^. Cytoskeletal contractility is governed by interactions between myosin and actin. Phosphorylation of myosin at Ser19 is the key regulatory step for actin-mediated Mg^2+^-ATPase activity which results in activation of the myosin head leading to cell contractility^30^. Up-regulation of Myosin Light Chain Kinase (MLCK) activity, one of the kinases specific to MLC, has been shown to compromise endothelial barrier integrity under different pathological conditions^31^,^32^.

Src is a non-receptor tyrosine kinase ubiquitously expressed in the cytoplasm of mammalian cells. Given its ability to interact with several substrates, Src is involved in regulation of a variety of cellular processes including adhesion, migration, and differentiation^33^. In the context of cell adhesion, previous studies have shown that Src can be activated directly or indirectly by integrins upon binding to extracellular matrix proteins such as fibronectin, by interactions with Receptor Protein Tyrosine Kinases (RPTK) (e.g. Platelet Derived Growth Factor receptor - PDGF receptor) and by G-protein Coupled Receptors (GPCR)^33^. Furthermore, Src can influence cytoskeleton remodelling upon integrin clustering at the cell membrane. However, the interplay between Src, cell-cell adhesion, and cell contractility in the context of tumor cell extravasation through the endothelium is not well understood.

Here, we sought to examine the relative roles of endothelial cell-cell adhesion and contractility during extravasation of metastatic melanoma cells through the endothelium. We hypothesized that metastatic cancer cells disrupt the endothelium and promote intercellular gaps between endothelial cells by initiating both endothelial cell adherens junction disassembly and contractility and we term this an “unzipping-pulling” model. To test this model, we first measured melanoma-induced gap formation between endothelial cells when both processes are active. Then, we blocked cell contractility or adherens junction disassembly individually and examined the impact on melanoma-induced gap formation. While using a co-culture model of metastatic melanoma cells with endothelial cells results in the largest effect on endothelial gap formation, it also confounds the analysis, as we show here, because multiple types of interactions take place and many signalling pathways are contributing at the same time. Therefore, to better characterize the triggers used by metastatic melanoma cells we isolated the signals activated by tumor cell secreted cytokines and by receptor-ligand interactions. Previous studies from our group showed that in contrast to non-invasive melanoma cells, metastatic melanoma cells secrete large amounts of Interleukin (IL)-8 and present the Very Late Antigen (VLA)-4 receptor on the surface of the plasma membrane^3^,^34^,^35^. Therefore, we also investigated the effect of cytokine signalling (IL-8) and receptor-ligand interactions through VLA-4 separately. Furthermore, using live-cell imaging of a Src Fluorescence Resonance Energy Transfer (Src-FRET) biosensor, we show that when co-cultured with endothelial cells, metastatic melanoma cells use cytokines and receptor-ligand interactions to induce activation of Src and VE-cadherin phosphorylation in endothelial cells. Blocking melanoma-mediated Src activation in endothelial cells abrogates VE-cadherin phosphorylation and breakdown of endothelial cell-cell junctions. In conclusion, our studies suggest that both endothelial cell contractility and adherens junction disassembly are necessary to disrupt the endothelial barrier, and play a central role in mediating intercellular gap formation.

## Results

### Metastatic melanoma cells induce intercellular gap formation in endothelial monolayers

Melanoma cells of different metastatic potentials may interact with and affect the endothelium in different ways. Thus, we sought to investigate the impact of non-metastatic WM35 and metastatic A2058 melanoma cells on the integrity of the endothelial barrier. The WM35 cell line was originally derived from a melanoma lesion at the Radial Growth Phase when presumably most of the melanoma cells have not acquired the ability to form metastases, while the A2058 cell line was derived from a metastatic lymph node^36^. A striking difference between WM35 and A2058 cell lines is that the former does not form tumors in xenograft models in mice, while the latter is capable of growing tumors in an immune-compromised mouse model^37^,^38^. Furthermore, previous studies from our group have characterized these melanoma cell lines based on cell invasiveness and adhesiveness and found that these characteristics correlate with metastatic potential^39^. Given the physiological differences between the two cell lines, here we use them as models of melanoma cells with different metastatic potential to contrast the effects they produce when interacting with the endothelium.

**Figure 1:**
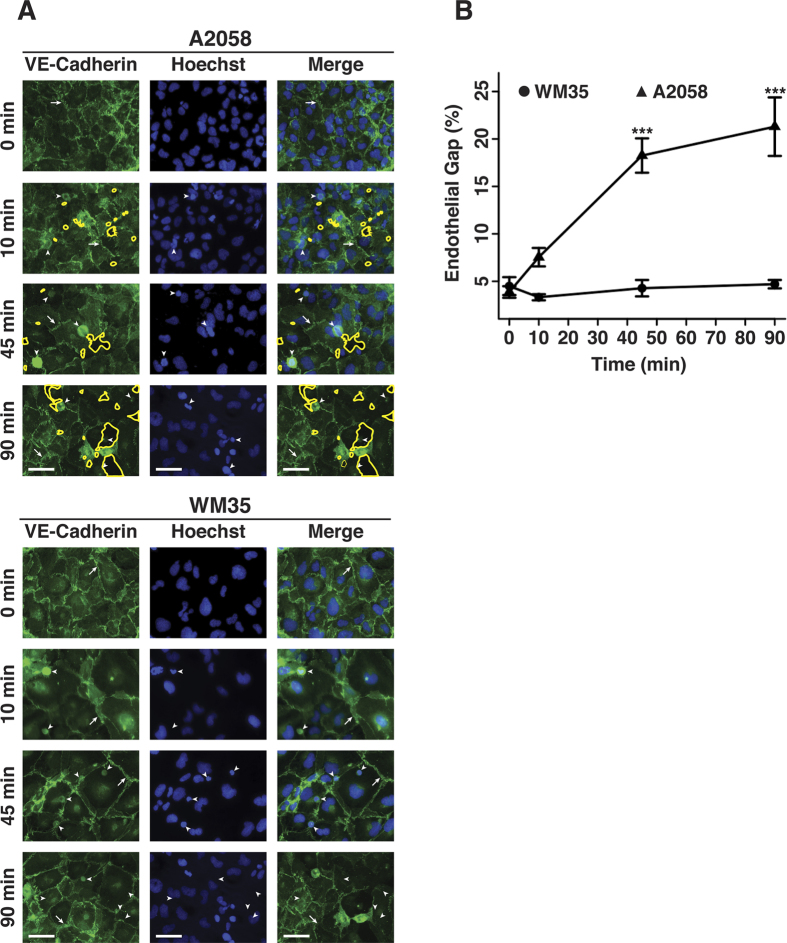
Metastatic melanoma cells induce gap formation between endothelial cells. (**A**) HPMEC monolayers cultured in direct contact with either A2058 (metastatic) or WM35 (non-metastatic) melanoma cells for 0, 10, 45 and 90 min. Endothelial cell junctions were immunostained with anti-VE-cadherin (green). Yellow outlines show intercellular gaps, arrows show adherens junctions and arrowheads show either A2058 or WM35 melanoma cells. Scale bars: 50 μm. (**B**) The percentage of endothelial gaps was quantified as the number of pixels within the gap regions divided by the number of pixels in the entire image. Results represent the mean +/−SEM (***p < 0.001 in comparison to monolayers treated with WM35 at equal times, n = 3).

To investigate whether melanoma cells of different metastatic potentials affect endothelial cell-cell junctions, A2058 and WM35 melanoma cells were co-cultured with human pulmonary microvascular endothelial cell (HPMEC) monolayers for specific periods of time. To visualize cell-cell junctions and gap formation, HPMEC monolayers were stained for VE-cadherin following culture in direct contact with either A2058 or WM35 melanoma cells for 10, 45 and 90 min (Fig. 1A). VE-cadherin staining showed the characteristic jagged pattern of adherens junctions where homodimers form across adjacent cell membranes. The percentage area of gaps between endothelial cells was used as a metric to compare endothelial cell-cell junction integrity as a function of treatment condition. Gap percentage was determined as the ratio of area within gaps between endothelial cells divided by the total area of each image. In the case of A2058 cells, after only 10 min of direct contact with endothelial cells, the gap percentage within the HPMEC monolayer increased in comparison to the negative control; at 45 and 90 min the effect was larger with gap percentages at 18% and 21% respectively, showing a time-dependency (Fig. 1B). In contrast, WM35 cells did not induce gap formation in HPMEC monolayers. These results are in agreement with previous studies that demonstrate that A2058 metastatic melanoma cells have the ability to induce gaps in endothelial monolayers in a time-dependent manner, while non-metastatic WM35 cells do not^3^,^40^,^41^. These results suggest that A2058 and WM35 melanoma cells interact with the endothelium through different mechanisms.

### Endothelial cell contractility and junction disassembly are necessary for gap formation: testing the unzipping-pulling model

The stability of endothelial cell-cell junctions and the maintenance of endothelial barrier integrity is thought to be regulated by a delicate balance between cell-cell adhesion and cell contractility. However, the relative roles of endothelial cell adhesion and contractility in the disassembly of endothelial cell-cell junctions in response to melanoma cells is not clear. We propose an unzipping-pulling model to describe breakdown of the endothelial barrier in response to cancer cells. Unzipping occurs due to the disruption of adhesion between VE-cadherin homodimers that hold the membranes of adjacent cells together (analogous to a zipper) while pulling results from endothelial cell contractility.

**Figure 2:**
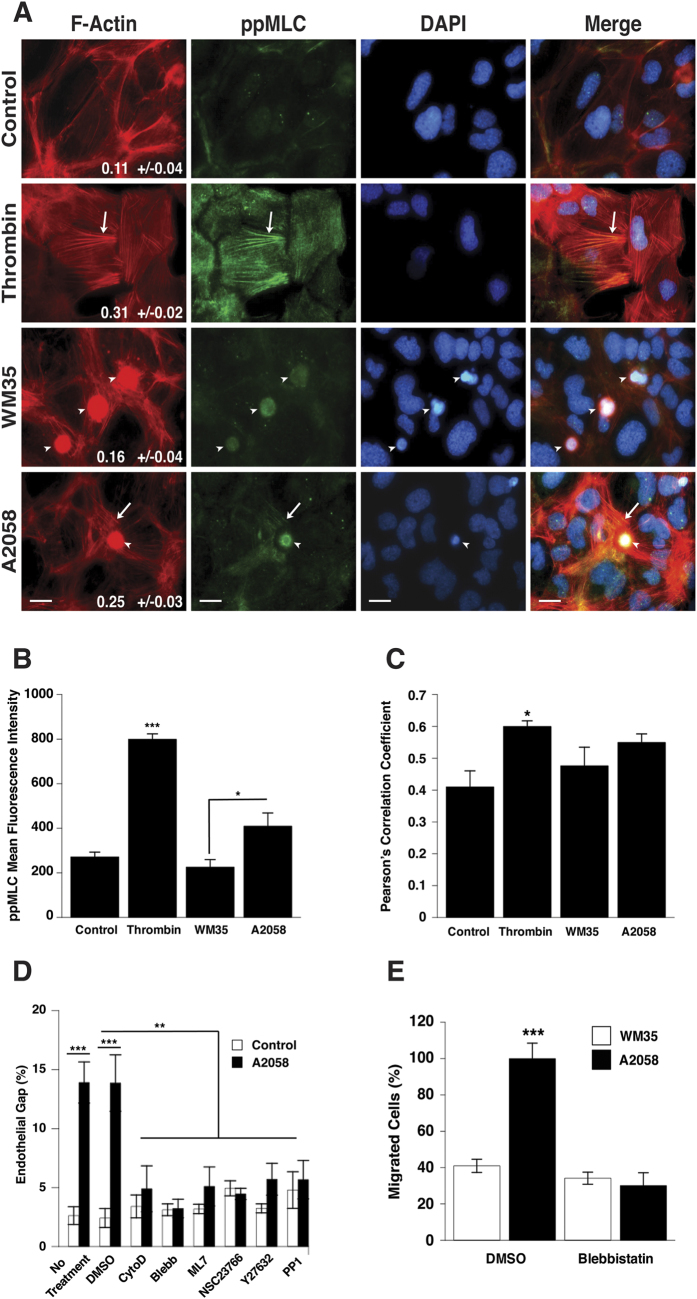
Endothelial cell contractility is necessary for melanoma-induced gap formation and subsequent endothelial barrier breakdown. (**A**) HPMEC monolayers stimulated with thrombin or cultured in direct contact with either A2058 (metastatic) or WM35 (non-metastatic) melanoma cells. Endothelial cell contractility was assessed via immunostaining of F-Actin (red) and ppMLC (green). Anisotropy numbers on F-actin images represent a measurement of fiber alignment (mean +/−SEM, n = 3). Scale bars: 10 μm. (**B**) Phosphorylation levels of MLC were determined by measuring mean fluorescence intensity of ppMLC images. Results represent the mean +/−SEM (n = 3). (**C**) Co-localization of ppMLC and F-actin filaments was measured from immunofluorescence images and is reported using Pearson’s correlation coefficient. Results represent the mean +/−SEM (n = 3). (**D**) HPMEC monolayers were pre-treated with inhibitors of contractility for 30 min and were then co-cultured in direct contact with A2058 melanoma cells for 45 min. Endothelial cell junctions were immunostained with anti-VE-cadherin and gap formation was quantified as the number of pixels within the gap regions over the number of pixels in the entire image. Results represent the mean ± SEM. A significant difference was found for the A2058 group between no treatment or DMSO and all the inhibitors. Also, significant differences were found between control and A2058 for no treatment and DMSO groups. No significant differences were observed between control and A2058 treated monolayers for any of the inhibitor treatments. (**E**) HPMEC monolayers cultured on 8 μm polycarbonate membranes were pre-treated with blebbistatin for 30 min and WM35 or A2058 melanoma cell migration across the monolayer was assessed. Results represent mean +/− SEM. (***p < 0.001, **p < 0.01, *p < 0.05, n = 3).

Previous studies have shown that VE-cadherin mediated cell-cell junctions act as mechanotransduction points in endothelial monolayers^11^,^42^. Local external force applied at these contacts triggers global signals that propagate throughout endothelial monolayers and gaps form at sites that are distant from the force application point. This requires a coupling mechanism with the actin cytoskeleton. Endothelial cell contractility can also exert force at cadherin-mediated contacts. To examine the role of endothelial contractility in disruption of endothelial cell-cell junctions in response to melanoma cells, first, we examined the formation of filamentous actin fibers and the levels of di-phosphorylated (pp)-MLC in endothelial cells in response to co-culture with melanoma cells. Image analysis was used to quantitate the anisotropy of fiber arrays in individual cells and was used as a proxy for actin stress fiber alignment. In agreement with previous studies^43^, results show that resting endothelial cells exhibit actin bundles located in the periphery (low anisotropy) and low levels of ppMLC (Fig. 2A,B). In contrast, endothelial cells stimulated with thrombin, a potent endothelial barrier disruptor, show high levels of ppMLC that co-localize with aligned actin stress fibers (high anisotropy) (Fig. 2C). Endothelial cells co-cultured with A2058 metastatic melanoma cells show increased levels of ppMLC compared to endothelial cells co-cultured with WM35 non-metastatic cells (Fig. 2B). Actin reorganization, evidenced by anisotropy measurements, as well as co-localization of ppMLC and actin stress fibers, is more evident in endothelial cells around A2058 cells than around WM35 cells, although no significant difference in the Pearson’s correlation coefficient between A2058 and WM35 cells was detected (Fig. 2B,C).

In addition, we treated endothelial cells with inhibitors of proteins that regulate cytoskeletal organization and cellular contractility. Monolayers of HPMECs were pre-treated with contractility inhibitors for 30 min, gently rinsed, and then immediately cultured in direct contact with A2058 metastatic melanoma cells. Decreasing the contractility of the endothelial cells by treatment with the actin polymerization inhibitor cytochalasin D, the non-muscle ATPase inhibitor blebbistatin^44^, the MLCK inhibitor ML-7^45^, the Rac1 inhibitor NSC23760, or the ROCK inhibitor Y27632^46^ abrogated A2058 melanoma cell-induced gap formation between endothelial cells (Fig. 2D). Gap formation between endothelial cells was reduced to the same levels as the control groups for all inhibitor treatments. Also, cell extravasation of A2058 metastatic melanoma cells across the endothelial barrier was significantly decreased when endothelial cell contractility was blocked using blebbistatin (Fig. 2E). In the absence of blebbistatin, migration of WM35 cells across the endothelial barrier was significantly lower than migration of A2058 cells; which is consistent with our previous result showing that non-metastatic melanoma cells do not disrupt the endothelium. These results suggest that actin reorganization and phosphorylation of MLC leading to cell contractility in the endothelium regulate gap formation, endothelial barrier breakdown and eventual migration of metastatic melanoma cells across the endothelium.

**Figure 3:**
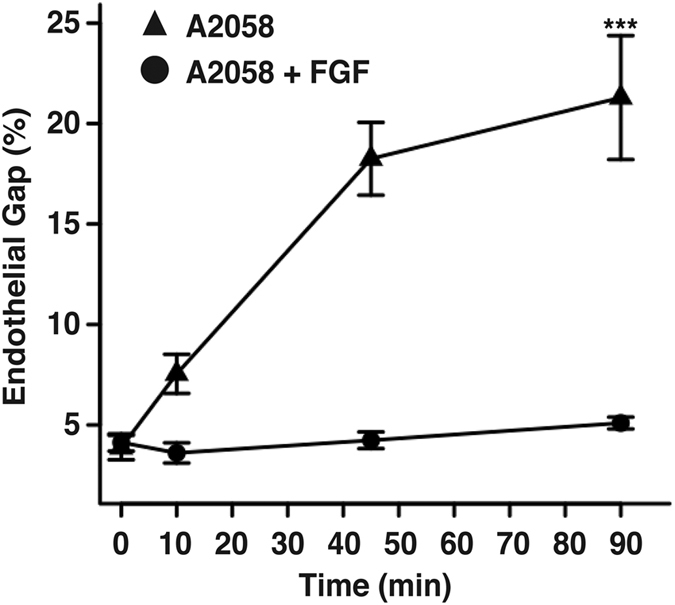
Stabilization of endothelial VE-cadherin junctions blocks melanoma-induced gap formation and subsequent endothelial barrier breakdown. HPMEC monolayers were treated with FGF1 for 1 hour and immediately cultured in direct contact with A2058 melanoma cells for 0, 10, 45 and 90 min. Endothelial cell junctions were immunostained with anti-VE-cadherin and gap formation was quantified as the number of pixels within gap regions over the number of pixels within the entire image. Results represent the mean +/−SEM, (***p < 0.001 in comparison to monolayers treated with FGF-1 at equal times, n = 3).

To study the contribution of VE-cadherin-mediated cell-cell adhesion on melanoma-induced junction disassembly, HPMEC monolayers were treated with fibroblast growth factor (FGF)-1 to artificially stabilize cell-cell junctions. Previous studies have shown that FGF-1 stimulation of endothelial cell monolayers promotes binding of VE-cadherin to p120-catenin; this interaction prevents cadherin internalization by blocking the binding site necessary for clathrin-mediated endocytosis thus stabilizing adherens junctions and enhancing endothelial barrier 47,48,49. Monolayers of HPMECs were treated with FGF-1 for 1 hour, gently rinsed, and then immediately co-cultured in direct contact with A2058 melanoma cells for 0, 10, 45, or 90 min. Melanoma-induced gap formation between endothelial cells was significantly reduced for FGF-1 treated monolayers in comparison to controls (Fig. 3 and Fig. S1). This data suggests that disruption of homodimer interactions of VE-cadherin across cell membranes is necessary for gap formation.

### Metastatic melanoma cells use multiple types of signals to trigger gap formation between endothelial cells

**Figure 4:**
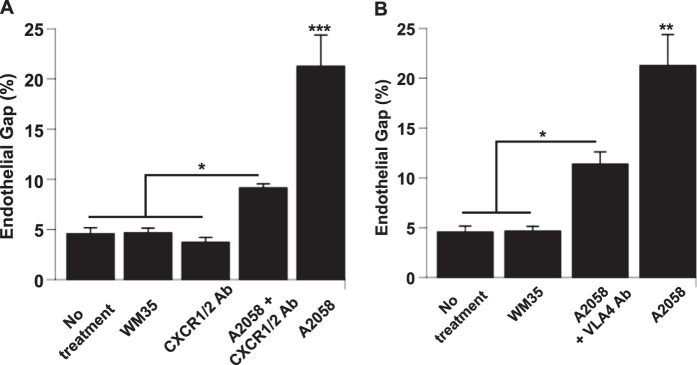
Metastatic melanoma cells use IL-8 signalling and VLA-4/VCAM-1 interactions to induce gap formation and subsequent endothelial barrier breakdown. (**A**) HPMEC monolayers were pre-treated with neutralizing antibodies against CXCR1 and CXCR2 receptors for 1 hour and immediately cultured in direct contact with A2058 melanoma cells for 90 min. Endothelial cell junctions were immunostained with anti-VE-cadherin and gap formation was quantified as the number of pixels within gap regions over the number of pixels within the entire image. (**B**) HPMEC monolayers were cultured in direct contact with either A2058 melanoma cells alone or A2058 melanoma cells pre-treated with VLA-4 neutralizing antibodies. Results represent the mean +/−SEM, (***p < 0.001, **p < 0.01, *p < 0.05, n = 3).

Melanoma-induced gaps between endothelial cells do not always form beneath the heterocellular contact; therefore, we hypothesized that multiple types of signalling contribute to gap formation, including receptor-ligand interactions and also paracrine signalling. Several studies have shown that metastatic melanoma cells secrete a number of cytokines that affect the behavior of the endothelium under physiological and pathological conditions. For instance, Gutova *et al*. analysed the media of metastatic A2058 melanoma cells using a cytokine array and found that IL-8 was the most abundant cytokine secreted, followed by Tissue Inhibitor of Metalloproteinase (TIMP)-2, Monocyte chemoattractant protein (MCP)-1 and Interleukin (IL)-6^50^. In our lab, a previous study showed a marked difference between IL-8 concentration in the media of A2058 metastatic melanoma cells (15 ng/ml) and WM35 non-metastatic melanoma cells (0.084 ng/ml)^3^. Here, to test if A2058 metastatic melanoma cells use signalling pathways through IL-8 receptors C-X-C chemokine receptor type (CXCR)-1 and C-X-C chemokine receptor type (CXCR)-2 to induce gap formation in endothelial monolayers, we used neutralizing antibodies against these receptors. HPMEC monolayers were pre-treated with neutralizing antibodies, gently washed and co-cultured with A2058 metastatic melanoma cells. Results show that endothelial gap formation is significantly reduced when the IL-8 signalling pathway is blocked; however, gap formation is not completely abolished (Fig. 4A and Fig. S2). This suggests that IL-8 is one of the major players in endothelial barrier disruption induced by metastatic melanoma cells, but it is not the only one.

**Figure 5:**
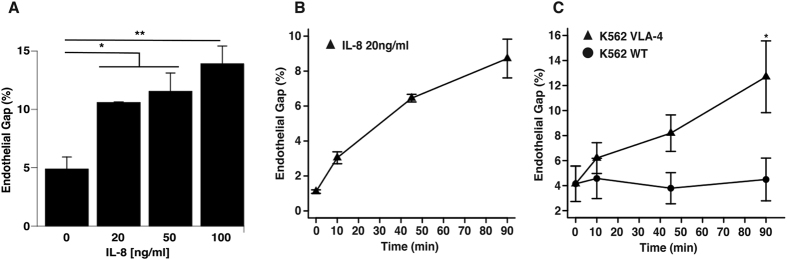
Isolated soluble factors and receptor-ligand interactions are sufficient to disrupt the endothelium and to form intercellular gaps. (**A**) Quantification of the percentage of gaps formed in HPMEC monolayers stimulated with increasing concentrations of IL-8 for 45 min. (**B**) Quantification of the percentage of gaps formed in HPMEC monolayers stimulated with 20 ng/ml IL-8 over a period of 0, 10, 45 and 90 min. (**C**) Quantification of the percentage of gaps formed in HUVEC monolayers stimulated with either K562-WT cells or K562-VLA-4 positive cells for 0, 10, 45 and 90 min. Statistical analysis was performed between K562-WT and K562-VLA-4 for equal incubation times. Plots represent the mean +/−SEM (*p < 0.05, **p < 0.01, n = 3).

To examine the impact of cytokines on gap formation in endothelial monolayers in the absence of receptor-ligand interactions between melanoma and endothelial cells, we tested the effect of IL-8 on HPMEC monolayers. IL-8 induced gap formation between HPMECs in a dose and time-dependent fashion (Fig. 5). Concentrations as low as 20 ng/ml of IL-8 were capable of inducing gaps between endothelial cells and disrupting the endothelial barrier; increasing concentrations of IL-8 promoted an increase in the percentage of gaps (Fig. 5A). Previous studies from our lab determined that A2058 melanoma cells secrete levels of IL-8 around 15 ng/ml over a period of 24 hours^3^. Thus, we also studied the effect of time given a physiological level of IL-8. For a constant concentration of IL-8, gap formation in HPMEC monolayers increased over time (Fig. 5B). The effect of IL-8 on gap formation between endothelial cells is smaller than when metastatic melanoma cells are co-cultured with endothelial cells (6% vs. 7% for 10 min, 11% vs. 18% for 45 min and 12% vs. 21% for 90 min). This is not surprising because melanoma cells can interact with endothelial cells in many ways and can trigger multiple pathways leading to gap formation at the same time as opposed to isolated activation of the CXCR1 and CXCR2 receptors by IL-8.

Previous studies have also shown that cancer cells can interact with the endothelium through receptor-ligand interactions^51^. Metastatic melanoma cells interact with the endothelium through binding interactions between the VLA-4 receptor and vascular cell adhesion molecule (VCAM)-1 displayed on the surface of endothelial cells^52^,^53^,^54^. Flow cytometry confirmed a higher expression of the VLA-4 receptor on A2058 metastatic melanoma cells compared to WM35 non-metastatic melanoma cells (Table 1). To test if A2058 metastatic melanoma cells interact with endothelial cells through VLA-4/VCAM-1 binding, we pre-treated A2058 cells with neutralizing antibodies against the VLA-4 receptors and immediately cultured them in direct contact with endothelial cells to assess gap formation. Results show that when the VLA-4/VCAM-1 interaction is blocked, gap formation in endothelial cells is significantly reduced (Fig. 4B). These results support our hypothesis that metastatic cancer cells use a combination of cytokine and receptor-ligand signalling pathways to disrupt the endothelial barrier.

To decouple the effects of VLA-4/VCAM-1 interactions from cytokine secretion on gap formation in endothelial monolayers, we performed experiments with K562 human erythroleukemia wild type (WT) cells and K562 cells stably transfected with the α4 integrin (K562 VLA-4). The functional VLA-4 receptor is formed via dimerization of the β1 and α4 integrin units. K562 WT cells normally express the β1 integrin and naturally lack the α4 integrin unit. In contrast, K562 cells transfected with the α4 integrin express a functional VLA-4 membrane receptor (Fig. S5A,B). In addition, cytokine secretion from K562 cells is low^55^. Co-culture of HPMEC monolayers with K562 VLA-4 cells resulted in an increase in gap formation between endothelial cells in comparison to co-culture with K562 WT cells (Fig. 5C). These results show that interaction of VLA-4 and VCAM-1 receptors on A2058 metastatic melanoma cells and endothelial cells, respectively, is sufficient to disrupt VE-cadherin interactions on adjacent endothelial cells to compromise the endothelial barrier.

### Endothelial cell gap formation induced by metastatic melanoma cells is mediated by Src activation

Src is a ubiquitous kinase present in the cytoplasm of mammalian cells that can phosphorylate multiple substrates^33^. We posited that A2058 metastatic melanoma cells could activate Src to subsequently phosphorylate VE-cadherin and disrupt the cadherin/catenin complex thereby inducing gap formation. To test this hypothesis, HPMEC monolayers were treated with PP1, a Src inhibitor, and gap formation between endothelial cells was quantified. Inhibition of Src reduced A2058-mediated gap formation to levels comparable to control monolayers (Last column Fig. 2D). This result suggests that Src activation and signalling within endothelial cells is necessary for melanoma-induced disruption of endothelial cell-cell junctions.

**Figure 6:**
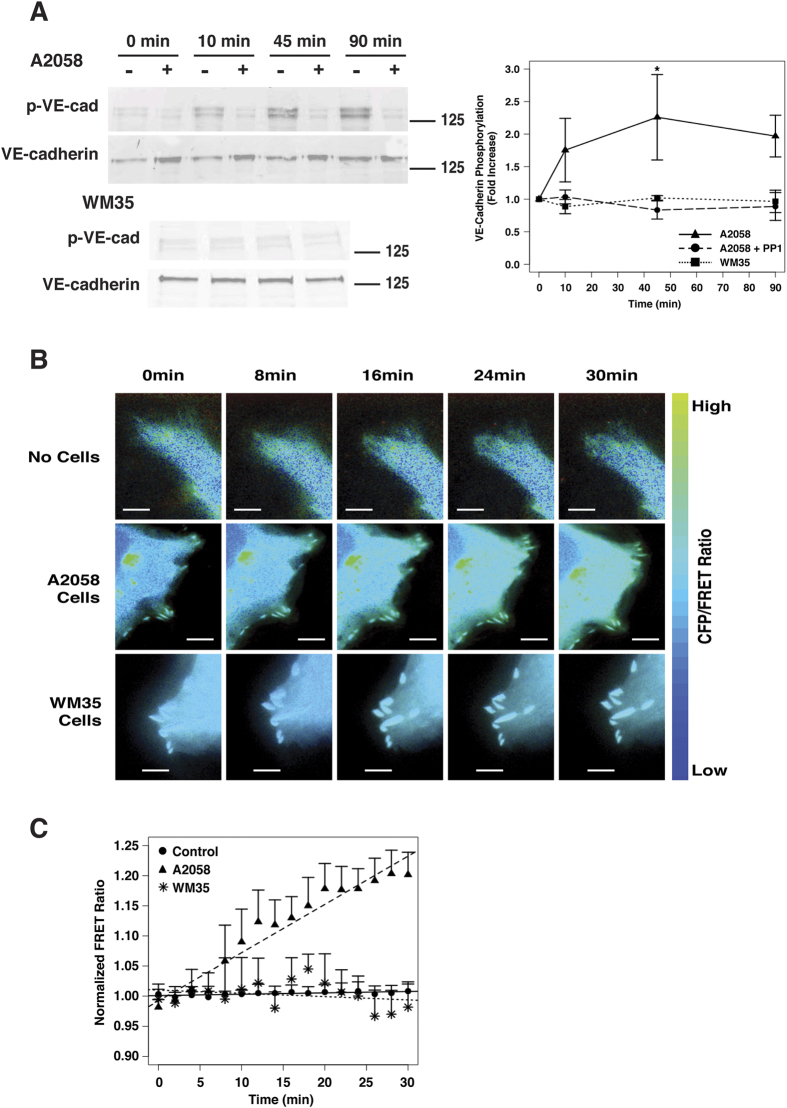
Metastatic melanoma cells induce VE-cadherin phosphorylation via Src activation. (**A**) Western blot and densitometric analysis of total and phosphorylated VE-cadherin in endothelial cells in co-culture with either A2058 or WM35 melanoma cells. Endothelial cells were either left untreated or pre-treated with the Src inhibitor PP1 prior to co-culture with melanoma cells. (**B**) Time-lapse images of endothelial cells expressing the Src FRET biosensor stimulated with either A2058 metastatic melanoma cells WM35 non-metastatic melanoma cells, or media only. Images are pseudocolored showing CFP/FRET ratio. Scale bars represent 5 μm. (**C**) Quantification of CFP/FRET ratio normalized to background signal (before stimulation) for each time-lapse image set and then normalized to negative control. Plot represent mean +/−SEM (n = 6).

Previous studies have suggested that Src can phosphorylate VE-cadherin, which can lead to an increase in vascular permeability^28^,^56^,^57^,^58^. Thus, we sought to determine whether A2058 melanoma cells could induce VE-cadherin phosphorylation in HPMECs. Endothelial monolayers were incubated for 0, 10, 45 and 90 min with A2058 metastatic melanoma cells and VE-cadherin phosphorylation was monitored via western blotting (Fig. 6A). A steady increase in VE-cadherin phosphorylation at site Y731 or Y658 was observed with time, with increased levels of phosphorylation observable after 10 min of co-culture with A2058 cells. In contrast, co-culture of HPMEC monolayers with non-metastatic WM35 cells did not promote VE-cadherin phosphorylation over the same time period. HPMECs were also treated with PP1 to determine whether Src activation mediates A2058-induced VE-cadherin phosphorylation. Treatment with PP1 reduced VE-cadherin phosphorylation to background levels. These results taken together suggest that metastatic melanoma cells can trigger Src activation, which induces VE-cadherin phosphorylation.

To further study induction of Src activation in endothelial cells by metastatic melanoma cells, we used a FRET biosensor that decreases FRET signal when Src is activated (Fig. 6B and Fig. S4A). HPMECs were transfected with the biosensor and were plated as monolayers. In agreement with endogenous Src localization, transfected cells showed localization of the sensor throughout the cytoplasm (Fig. S4B). Following stimulation, transfected cells were monitored for 30 min, images were taken every two minutes and two images were taken at each time-point (Donor/Donor-Cyan Fluorescent Protein (CFP) and Donor/Acceptor-FRET). The CFP/FRET ratio was used as an indicator of Src activity. Normalized CFP/FRET ratio results show that upon direct contact, metastatic melanoma cells activate Src in endothelial cells within minutes (Fig. 6C). In addition, compared to the negative control or endothelial cells in contact with WM35 non-metastatic melanoma cells, endothelial cells in co-culture with A2058 cells show a steady increase in normalized CFP/FRET ratio with time.

### Src activation in endothelial cells is triggered by IL-8 and by stimulation of VCAM-1

**Figure 7:**
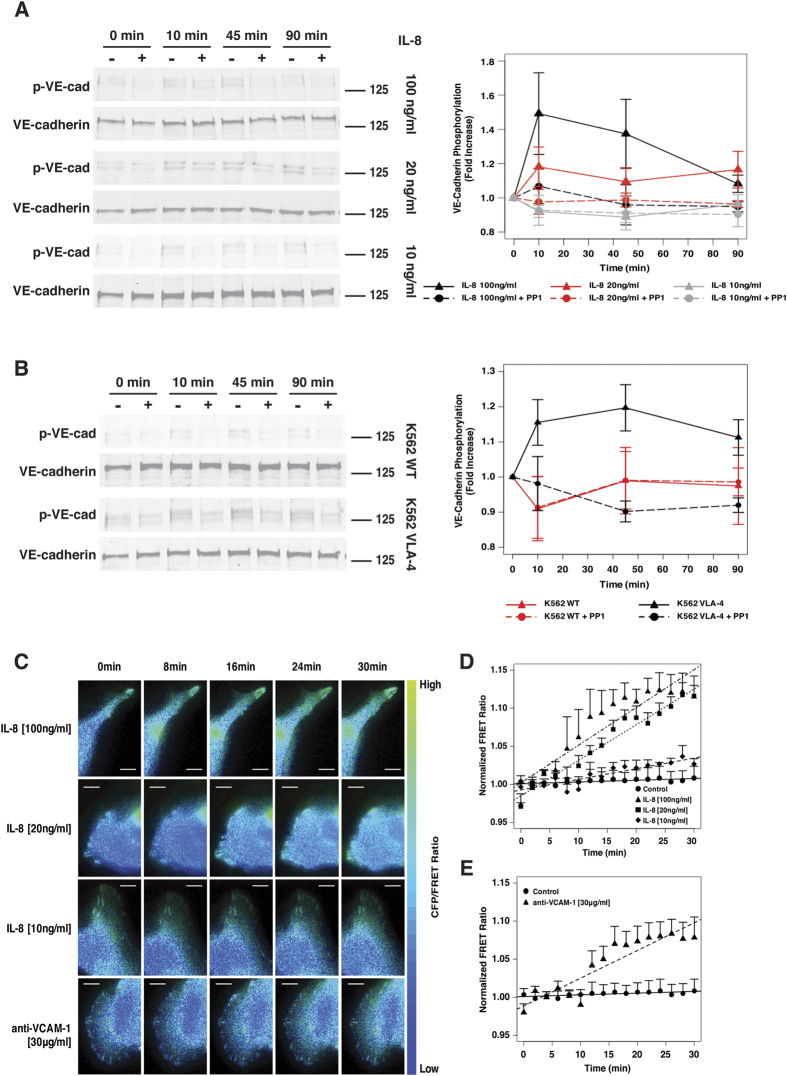
Soluble and receptor-ligand interactions activate Src in endothelial cells resulting in VE-cadherin phosphorylation. (**A**) Western blot and densitometric analysis of total and phosphorylated VE-cadherin induced by IL-8 [100, 20 or 10 ng/ml] after 0, 10, 45 and 90 min. (**B**) Western blot and densitometric analysis of total and phosphorylated VE-cadherin induced by K562-WT or K562-VLA-4 cells after 0, 10, 45 and 90 min. (**C**) Time-lapse images of endothelial cells expressing the Src FRET biosensor stimulated with different concentrations of IL-8 [100, 20 or 10 ng/ml] or with anti-VCAM-1 antibody [30 μg/ml]. Images are pseudocolored showing CFP/FRET ratio (Bars represent 5 μm). (**D,E**) Quantification of CFP/FRET ratio normalized to background signal (before stimulation) for each time-lapse image set and then normalized to negative control. Plots represent mean +/−SEM (n = 6).

Given that metastatic melanoma cells interact with the endothelium via cytokines and receptor-ligand binding, we sought to determine how these two signalling mechanisms independently regulate activation of Src within endothelial cells. To confirm that IL-8 can activate Src and can lead to phosphorylation of VE-cadherin in HPMECs, we examined VE-cadherin phosphorylation levels as a function of IL-8 concentration and treatment time by western blotting. HPMEC monolayers were stimulated with 10, 20, or 100 ng/ml of IL-8 for 10, 45 and 90 min (Fig. 7A). VE-cadherin phosphorylation levels increased with time and with IL-8 concentration. Treatment of HPMEC monolayers with PP1 prior to treatment with IL-8 blocked VE-cadherin phosphorylation. Furthermore, co-culture of HPMEC monolayers with K562 VLA-4 expressing cells resulted in increased phosphorylation of VE-cadherin in comparison to co-culture with control K562 WT cells (Fig. 7B). Pre-treatment of the HPMEC monolayers with PP1 abrogated VLA-4/VCAM-1 induced VE-cadherin phosphorylation. These data suggest that IL-8 and VLA-4/VCAM-1 interactions can induce VE-cadherin phosphorylation via activation of Src.

To further examine activation of Src in endothelial cells by IL-8 and VLA-4/VCAM-1 interactions, endothelial cells were transfected with the Src FRET biosensor and changes in FRET signal upon stimulation with different concentrations of IL-8 or with anti-VCAM-1 antibodies were monitored (Fig. 7C). Computation of the normalized CFP/FRET ratio revealed that IL-8 and VCAM-1 can activate Src within minutes of stimulation in comparison to control treated samples (no stimulation) (Fig. 7D,E). Although different concentrations of IL-8 (10, 20 and 100 ng/ml) were used to treat endothelial monolayers containing cells transfected with the Src FRET biosensor, only the 20 and 100 ng/ml IL-8 concentrations were sufficient to activate Src (Fig. 7D). There was no difference between the group treated with 10 ng/ml of IL-8 and the negative control where no cytokine was used. These results show that IL-8 activates Src in endothelial cells in a concentration and time dependent manner. Previously, Allingham *et al*. showed that engagement of the Intercellular adhesion molecule (ICAM)-1 receptor by monocytic THP-1 cells ultimately results in phosphorylation of VE-cadherin via Src activation^57^. Here, we show that similarly to ICAM-1, activation of the VCAM-1 receptor induces Src activation leading to VE-cadherin phosphorylation.

## Discussion

In the present study we examined the signalling mechanisms by which metastatic melanoma cells induce intercellular gap formation between endothelial cells. We find that metastatic melanoma cells initiate disruption of endothelial cell-cell junctions and promote the formation of intercellular gaps in endothelial monolayers in as short as 10 min of direct co-culture. In contrast, non-metastatic melanoma cells do not disrupt the endothelium after direct contact for longer periods of time. A major difference between metastatic and non-metastatic melanoma cells is the ability of the former to interact with the endothelium via secretion of cytokines and receptor-ligand interactions which can mediate transendothelial migration^7^. Multiple studies from our lab and others have shown that only interactions between cell lines derived from metastatic tumors result in endothelial barrier breakdown, whereas non-metastatic cancer cells do not disrupt the endothelium^3^,^34^,^59^.

In an effort to characterize the contribution of endothelial cell contractility to melanoma-induced endothelial barrier breakdown, we analysed changes in stress fiber formation and phosphorylation of MLC in endothelial cells in response to thrombin and interactions with A2058 metastatic and WM35 non-metastatic melanoma cells. This analysis revealed that thrombin, as previously shown^43^, increased phosphorylation of MLC and formation of F-actin stress fibers in endothelial cells. Similarly, upon contact with A2058 metastatic melanoma cells, F-actin fibers in endothelial cells are rearranged, phosphorylation of MLC increases and co-localization of ppMLC with stress fibers increases, although the difference is not significant when compared to control and WM35-treated monolayers. Conversely, co-culture with WM35 non-metastatic melanoma cells did not increase reorganization of F-actin stress fibers in endothelial cells and did not induce MLC phosphorylation. These results suggest that metastatic A2058 melanoma cells induce cytoskeletal re-arrangement in endothelial cells leading to increased cell contractility. In addition, we do find that inhibition of cytoskeletal contractility within endothelial cells blocked melanoma-induced gap formation suggesting that endothelial cell contractility is indeed necessary for intercellular gap formation. Furthermore, migration of A2058 metastatic melanoma cells was significantly decreased when endothelial cell contractility was blocked in a transwell study.

The present results further demonstrate the importance of VE-cadherin disassembly for intercellular gap formation. Due to redundancy of Fibroblast Growth Factors (FGFs) and the lethal nature of knocking-out their receptors, the physiological role of the FGF system has been difficult to elucidate^48^. By using viral mediated systemic expression of soluble traps that capture FGFs in circulation with different affinities, Murakami *et al*. were able to show that FGF signalling is required for maintenance of adherens junctions and the integrity of the endothelial barrier. Treatment of endothelial monolayers with FGF-1 showed a slight increase in impedance, which correlates with decreased permeability. Also, FGF-1 treatment was sufficient to prevent diffusion of VE-cadherin from cell-cell contacts into the cytosol after incubation with VEGF-A^47^. In our study, treatment with FGF-1 to lock VE-cadherin homodimers at cell-cell junctions prevented melanoma-mediated gap formation in endothelial monolayers.

Based on our results showing that A2058 melanoma cells use IL-8 to induce gap formation between endothelial cells and previous results showing that metastatic melanoma cells release IL-8^34^,^50^,^60^, we tested the ability of IL-8 cytokine stimulation alone to induce gap formation between endothelial cells. The higher the concentration of IL-8 or the longer the endothelial cells were stimulated with IL-8, the larger the effect on disruption of cell-cell junctions. Using similar concentrations of IL-8, previous studies have shown that stimulation of endothelial cells with IL-8 results in cytoskeletal rearrangement *in vitro*^61^ and also increased permeability of the endothelial barrier through activation of VEGFR in a VEGF-independent mechanism. This effect has also been further confirmed *in vivo*^62^. Interestingly, the results of our studies do not show a plateau trend either at high concentrations (50, 100 ng/ml) or long incubation times (45, 90 min) of IL-8, suggesting the effect of metastatic cancer cells on endothelial cell barrier could potentially be greater if the cancer cells are in close proximity to the endothelium for longer or if local concentrations of cytokines secreted by melanoma cells reach higher levels than the ones tested here.

Physical interactions through membrane receptors between metastatic cancer cells and the endothelium are also very important for transendothelial migration during extravasation. Although, circulating cancer cells need to be arrested to interact with the endothelium, here we show that these interactions are not only important to capture cancer cells from the blood stream but also contribute to endothelial junction disassembly. Similar to our results here, previous studies have shown that the high affinity interaction between the VLA-4 and the VCAM-1 receptor enhances migration of melanoma cells across the endothelium and also that higher adhesiveness between cancer and endothelial cells is correlated with higher metastatic potential^35^,^54^.

After establishing the disruptive effect of metastatic melanoma cells on endothelial monolayers and identifying IL-8 and VLA-4/VCAM-1 interactions as important triggers, we turned our attention to examining mechanistically how these tumor cell signals promote disruption of endothelial junctions. Src is a kinase known to regulate cytoskeleton interactions with integrins in the cell membrane and to phosphorylate membrane receptors that mediate cell adhesion^63^. Previously, Allingham *et al*. showed that in the context of leukocyte TEM, VE-cadherin is phosphorylated by Src via ICAM-1 signalling^57^. Also, Src has been shown to be a mediator in disrupting the endothelial barrier and can potentiate cancer cell extravasation in response to VEGF treatment^56^. Here, using a Src FRET biosensor, we monitored the activation of Src in live endothelial cells in response to tumor cell signals. When endothelial cells directly contacted metastatic melanoma cells, Src in endothelial cells was activated within minutes of the initial contact. Western blot analyses further showed that metastatic melanoma cell-induced Src activation in endothelial cells correlated with phosphorylation of VE-cadherin. In contrast, non-metastatic melanoma cells in direct contact with endothelial cells did not induce phosphorylation of VE-cadherin. Furthermore, Src inhibition by treatment of endothelial monolayers with PP1 was sufficient to abrogate metastatic melanoma-mediated gap formation between endothelial cells. Together, these data suggest that activation of Src in endothelial cells by metastatic melanoma cells is necessary to break the endothelial barrier.

Overall, the results presented show that metastatic melanoma cell signals including soluble cytokines and direct receptor-ligand interactions can impact endothelial cell contractility and adherens junction stability thereby mediating endothelial barrier disruption. Based on our results and in agreement with previous work suggesting that tyrosine phosphorylation of VE-cadherin through Src activation is not sufficient to decrease barrier function of the endothelium^28^, we propose that in fact, both cell contractility and adherens junction disassembly are necessary for endothelial barrier breakdown. Further studies on the interplay between endothelial cell contractility and cell-cell adhesion in response to tumor cell signals will permit a clearer view of how biochemical cues and mechanical forces act in concert to facilitate extravasation and may suggest new targets or approaches for preventing metastasis.

## Materials and Methods

### Cell Culture

Human pulmonary microvascular endothelial cells (HPMECs) were a generous gift from Dr. Kirkpatrick from the Institute of Pathology in Johannes Gutenberg University, Germany. Human umbilical vein endothelial cells (HUVECs) and A2058 cells were purchased from ATCC. K562-WT and K562-VLA-4 cells were a generous gift from Dr. Martin Humphries from the University of Manchester, England. WM35 cells were kindly provided by Dr. Meenhard Herlyn, from the Wistar Institute in Philadelphia, PA. HPMECs were cultured in 199 Media supplemented with 10% Fetal Bovine Serum (FBS; Atlanta Biologicals), 2 mM L-Glutamine, 100 U/ml Penicillin and 100 μg/ml Streptomycin. HUVECs were cultured in F12-K Media supplemented with 10% FBS, 30 μg/ml Endothelial cell growth supplement (ECGS; Corning), 50 μg/ml Heparin (Alfa Aesar), 100 U/ml Penicillin, 100 μg/ml Streptomycin. WM35, K562 WT cells and K562 VLA-4 expressing cells were cultured in RPMI Media supplemented with 10% FBS, 100 U/ml Penicillin, 100 μg/ml Streptomycin. For K562 VLA-4 expressing cells, 1 mg/ml G418 was also included. A2058 melanoma cells were cultured in DMEM Media supplemented with 10% FBS, 2 mM L-Glutamine, 100 U/ml Penicillin and 100 μg/ml Streptomycin. All cell lines were maintained in a humidified incubator at 37 °C and 5% CO_2_. Media formulations, supplements, antibodies and reagents were purchased from Invitrogen unless otherwise stated.

### Immunofluorescence for gap experiments

HPMECs were grown on top of 25 mm round No.1 cover glass coated with fibronectin (10 μg/ml; Corning). Once HPMECs formed confluent monolayers, endothelial cells were incubated with either A2058 or WM35 melanoma cells, K562 WT cells or K562 VLA4 expressing cells in direct contact for 0, 10, 45 and 90 minutes. After experiments, monolayers of HPMECs were fixed in 4% formaldehyde for 15 min at room temperature. Cells were then treated with a permeabilization/blocking buffer containing 5% Goat Serum and 0.3% Triton X-100 in 1× phosphate buffered saline (PBS; R&D Systems) for 1 hour at room temperature. Cells were then incubated with a primary antibody for VE-cadherin (1:300 dilution; Cell Signaling), in an antibody dilution buffer (ADB) containing 1% bovine serum albumin (BSA) and 0.3% Triton X-100 in PBS over night at 4 °C. Cells were then washed three times in PBS and incubated with a fluorescently-tagged goat anti-mouse secondary antibody (1:1000 dilution; Invitrogen), in ADB for 1 hour at room temperature protected from light. Cells were then washed three times in PBS and counterstained with Hoechst as per manufacturer’s instructions. Finally, cover glass was mounted on microscope slides using Fluoromount-G (Southern Biotech) before imaging. Images were acquired using a Nikon Eclipse Ti-E inverted microscope equipped with a Photometrics CoolSNAP HQ^2^ CCD camera and a 100× oil objective. Using a grid of 6 by 4, a total of 24 images were captured per cover glass. Gap percentage measurements were determined as the area not covered by HPMECs (gaps) divided by the total area of each image. Analysis was performed using Image J software. Final results are reported as the mean ± SEM of three independent replicates.

### Cytoskeletal analysis

Once HPMECs formed confluent monolayers, endothelial cells were incubated with either Thrombin for 10 minutes, A2058 or WM35 melanoma cells for 45 minutes. Cells were fixed and stained as described above. Specifically, cells were stained using a primary antibody for ppMLC (1:200 dilution; Thermo Scientific), followed by phalloidin (1:50 dilution; Sigma) for 30 minutes at room temperature and counterstained with Hoechst as per manufacturer’s instructions (Invitrogen). Images were acquired using a Nikon Eclipse Ti-E inverted microscope equipped with a Photometrics CoolSNAP HQ^2^ CCD camera and a 60 × oil objective. Anisotropy index was quantified using the FibrilTool plugin for Image J software^64^. At least 15 individual cells were analysed per treatment condition, and anisotropy indexes were averaged. Final results are reported as the mean+/− SEM of three independent experiments. To determine phosphorylation levels of MLC, the mean fluorescence intensity of individual cells was measured using Image J. At least 15 individual cells were measured per treatment condition and the results were reported as the mean +/−SEM of three independent experiments.

Co-localization of phosphorylated MLC and actin fibers in endothelial cells was assessed using the Colocalization plug-in for Image J software^65^. Pearson’s correlation coefficient was used as an index for co-localization of ppMLC and f-actin. At least 15 individual cells were measured per treatment conditions and the results were reported as mean +/−SEM of three independent experiments.

### Pharmacological Inhibition Studies

Where indicated, prior to A2058 co-culture, HPMEC monolayers were incubated with the following pharmacological inhibitors for 30 min: ML-7 (5 μM; Sigma); Blebbistatin (Blebb; 25 μM; Sigma); Cytochalasin D (CytoD; 0.01 μg/ml; Tocris Bioscience); Y27632 (5 μM; Tocris Bioscience); NSC23766 (200 μM; Tocris Bioscience); PP1 (0.17 μM; Sigma). All inhibitors were reconstituted in dimethyl sulfoxide (DMSO; Sigma). After the 30 min incubation, monolayers were gently washed with 2% FBS supplemented endothelial cell culture media followed by co-culture with A2058 melanoma cells as described above.

Fibroblast Growth Factor (FGF; 100 pg/ml) was reconstituted in PBS. Endothelial monolayers were incubated with FGF for 1 h. After the 1 h incubation, monolayers were gently washed with 2% FBS supplemented endothelial cell culture media followed by co-culture with A2058 melanoma cells.

Neutralizing antibodies against receptors CXCR1/2 (10 μg/ml) and integrins α4 and β1 (10 μg/ml) were reconstituted in sterile PBS. For inhibition of the IL-8 pathway, prior to co-culture with A2058 cells, endothelial cells were incubated with CXCR1/2 antibodies. For inhibition of the VLA-4/VCAM-1 pathway, prior to co-culture with endothelial cells, A2058 cells were incubated with α4 and β1 antibodies. In both cases, after 1 hour cells were gently washed and direct co-culture experiments were performed.

### Cell Migration

HPMECs were grown on polycarbonate membranes with 8 μm pore size coated with fibronectin (30 μg/ml) for 1 week, until confluent monolayers were formed. Before experiments, monolayers were either treated with Blebbistatin (25 μM; Sigma) for 30 min or left alone; A2058 melanoma cells were labelled with CMFDA tracking dye (1 μM; Invitrogen) for visualization and quantification purposes. A2058 melanoma cells were allowed to migrate for 4 hours. Image quantification was done using particle analysis on ImageJ. Results were expressed as a percentage of migrated cells normalized to the control group (DMSO alone).

### Fluorescence Resonance Energy Transfer (FRET)

The Src FRET biosensor was generously provided by Dr. Peter Yingxiao Wang from the University of California at San Diego (UCSD). The design of the Src FRET biosensor was described previously^66^ and it is briefly described here (Fig. S4). HPMECs were transfected overnight using Mirus 2020. Cells were allowed to recover for 1 day and then trypsinized and plated on top of 25 mm round No.1 cover glass coated with fibronectin (10 μg/ml). Monolayers were allowed to form for 2 days before performing FRET experiments. A Nikon Eclipse Ti-E inverted microscope equipped with a Photometrics CoolSNAP HQ^2^ CCD camera and a 60× oil objective was used for image acquisition. Two sets of filters were used to capture images of donor-donor (CFP image) and donor-acceptor (FRET image) at each time point. An excitation 426–446 nm bandpass filter and an emission 460–500 nm bandpass filter were used for CFP images; for FRET images only the emission filter was changed to a 520–550 nm bandpass filter. In both cases, a 455 nm long-pass dichromatic mirror was used to filter out excitation wavelengths. A live cell environmental control chamber was used to maintain the temperature at 37 °C, 5% CO_2_ and 75% humidity for live cell imaging. In addition, three sets of ND filters (ND4, ND8, ND16) were used to decrease photodamage to live cells. Images were acquired every 2 min for a period of 30 min. Initially, images of non-stimulated HPMECs were used to establish the signal background; cells were then stimulated either by direct contact with A2058 melanoma cells, IL-8 (10, 20 and 100 ng/ml), or anti vascular adhesion molecule (VCAM)-1 antibody (30 μg/ml, R&D Systems). Images were analysed using Image J and Fluocell software. Fluocell software was generously provided by Dr. Peter Yingxiao Wang and Dr. Kathy Shaoying Lu from UCSD; the results were calculated as a ratio of CFP/FRET at each time point normalized to the background signal.

### Flow Cytometry

Expression of the Very Late Antigen-4 (VLA-4) receptor on the cell membrane of A2058 melanoma cells, K562 WT cells, and K562 VLA-4 expressing cells was determined using flow cytometry. In brief, cells were incubated with either anti-α4 or anti-β1 integrin (1:100 dilution; R&D Systems) in a solution containing 0.1% BSA for 2 hours on a rocker with ice. Cells were then washed three times with PBS and further incubated with the secondary antibody (1:1000 dilution; Invitrogen) for 2 hours on a rocker with ice. Finally, cells were washed three times and run through the flow cytometer. Analysis of the data was performed using Flow Jo.

### Western Blot

Discontinuous 10% and 15% polyacrylamide gels were polymerized in house. Protein samples were run through the gels at a constant voltage of 125 V. Proteins were transferred onto a nitrocellulose membrane overnight using a wet tank at 4 °C. Proteins were then detected using primary antibodies for VE-cadherin (1:1000 dilution; Cell Signalling) and p-VE-cadherin (1:1000 dilution; Invitrogen) in a 1% BSA/TBST buffer overnight and incubation with a secondary goat anti-rabbit (1:10,000 dilution; Licor 800CW). Immunoblot detection was performed using an Odyssey fluorescent imager.

### Statistical Analysis

One-way ANOVAs with Tukey post hoc tests were used to determine significant differences in endothelial gap formation among indicated groups.

## Additional Information

**How to cite this article:** Aragon-Sanabria, V. *et al*. VE-Cadherin Disassembly and Cell Contractility in the Endothelium are Necessary for Barrier Disruption Induced by Tumor Cells. *Sci. Rep.* **7**, 45835; doi: 10.1038/srep45835 (2017).

**Publisher's note:** Springer Nature remains neutral with regard to jurisdictional claims in published maps and institutional affiliations.

## Supplementary Material

Supplementary Information

## Figures and Tables

**Table 1:** VLA-4 expression in WM35 and A2058 cell lines assessed by flow cytometry.

*VLA-4 Expression*		
Cell Line	α4 (Median AFU)	β1 (Median AFU)
WM35	100	100
A2058	150	132

Results are normalized to integrin expression in WM35 cell line.
