# Supplementary material for: VE-Cadherin Disassembly and Cell Contractility in the Endothelium are Necessary for Barrier Disruption Induced by Tumor Cells
Source: Sci Rep. 2017 Apr 10;7:45835. doi: 10.1038/srep45835 (PMC5385522; doi:10.1038/srep45835)
Supplement: Supplementary Information [file srep45835-s1.pdf]

VE-Cadherin Disassembly and Cell Contractility in the Endothelium are Necessary for Barrier Disruption Induced by Tumor Cells

Virginia Aragon-Sanabria[1], Steven E. Pohler[2], Vikram J. Eswar[1], Matthew Bierowski[2], Esther W. Gomez[1,2], Cheng Dong[1]

Supplementary Information

Figure S1

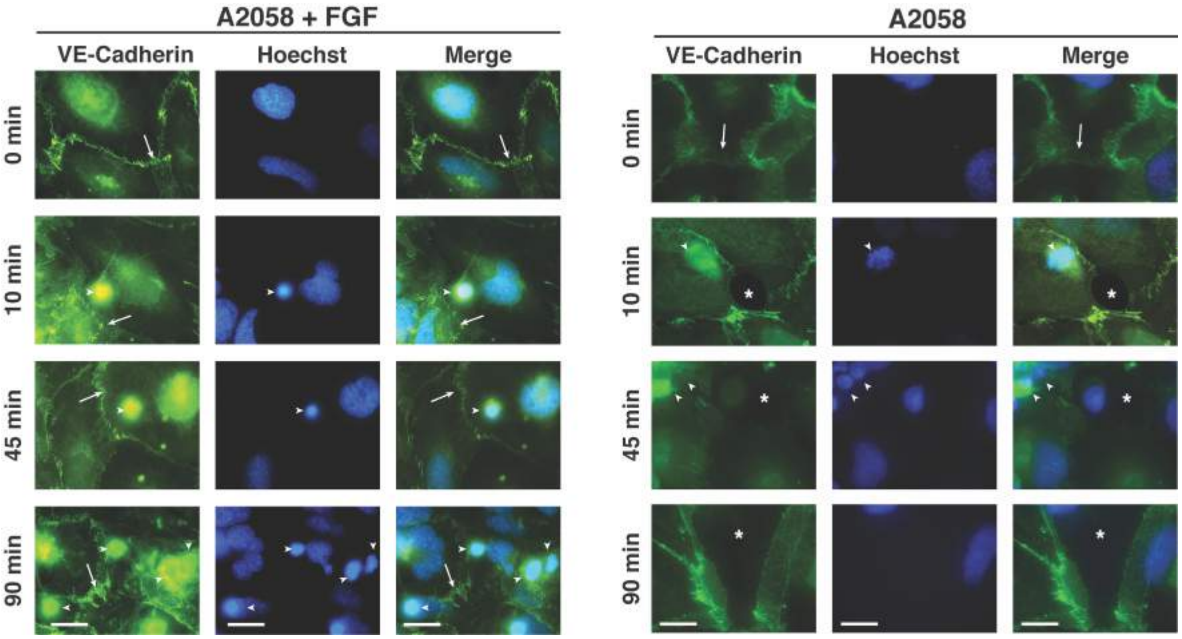

Supplemental Fig S1. Representative images of HPMEC cells either left alone or treated with FGF prior to co-culture with A2058 melanoma cells. Arrows show adherens junctions, arrowheads show A2058 melanoma cells and asterisks show gaps. Scale bars represent 10 μm.

Figure S2

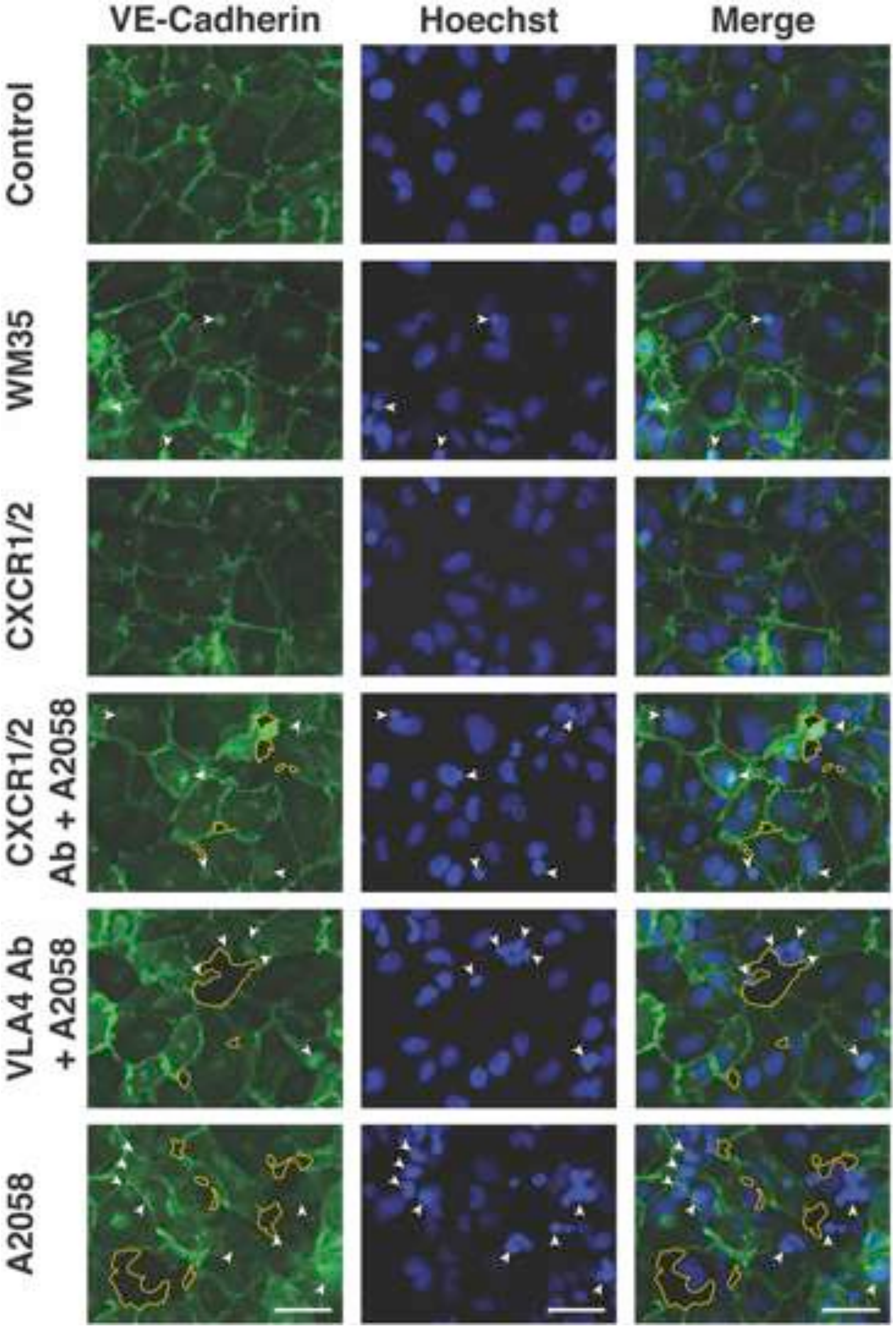

Supplemental Fig S2. Representative images of HPMEC cells either left alone or treated with WM35, CXCR1/2 antibodies, CXCR1/2 antibodies prior to co-culture with A2058 melanoma cells, or A2058 treated with VLA4 antibodies. Arrowheads show A2058 or WM35 melanoma cells. Gaps are outlined in yellow. Scale bars represent 50 µm.

Figure S3

A

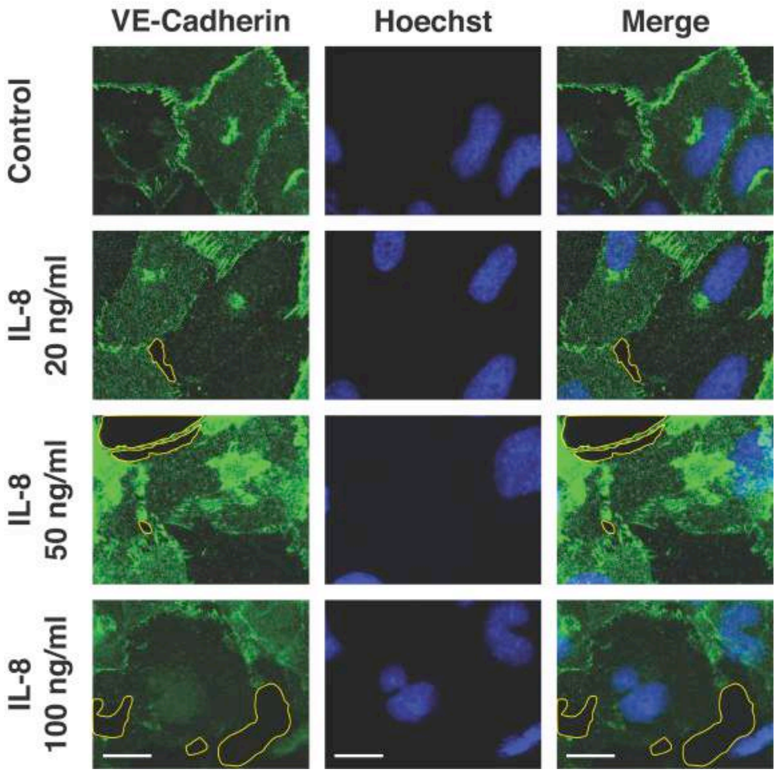
VE-Cadherin
Hoechst
Merge
Control
IL-8 20 ng/ml
IL-8 50 ng/ml
IL-8 100 ng/ml

B

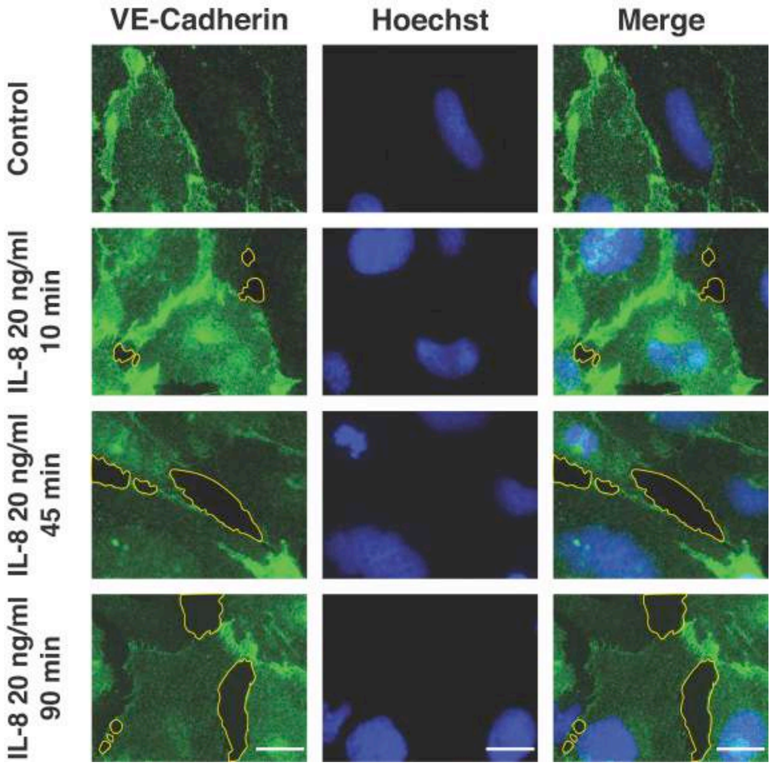
VE-Cadherin
Hoechst
Merge
Control
IL-8 20 ng/ml 10 min
IL-8 20 ng/ml 45 min
IL-8 20 ng/ml 90 min

C

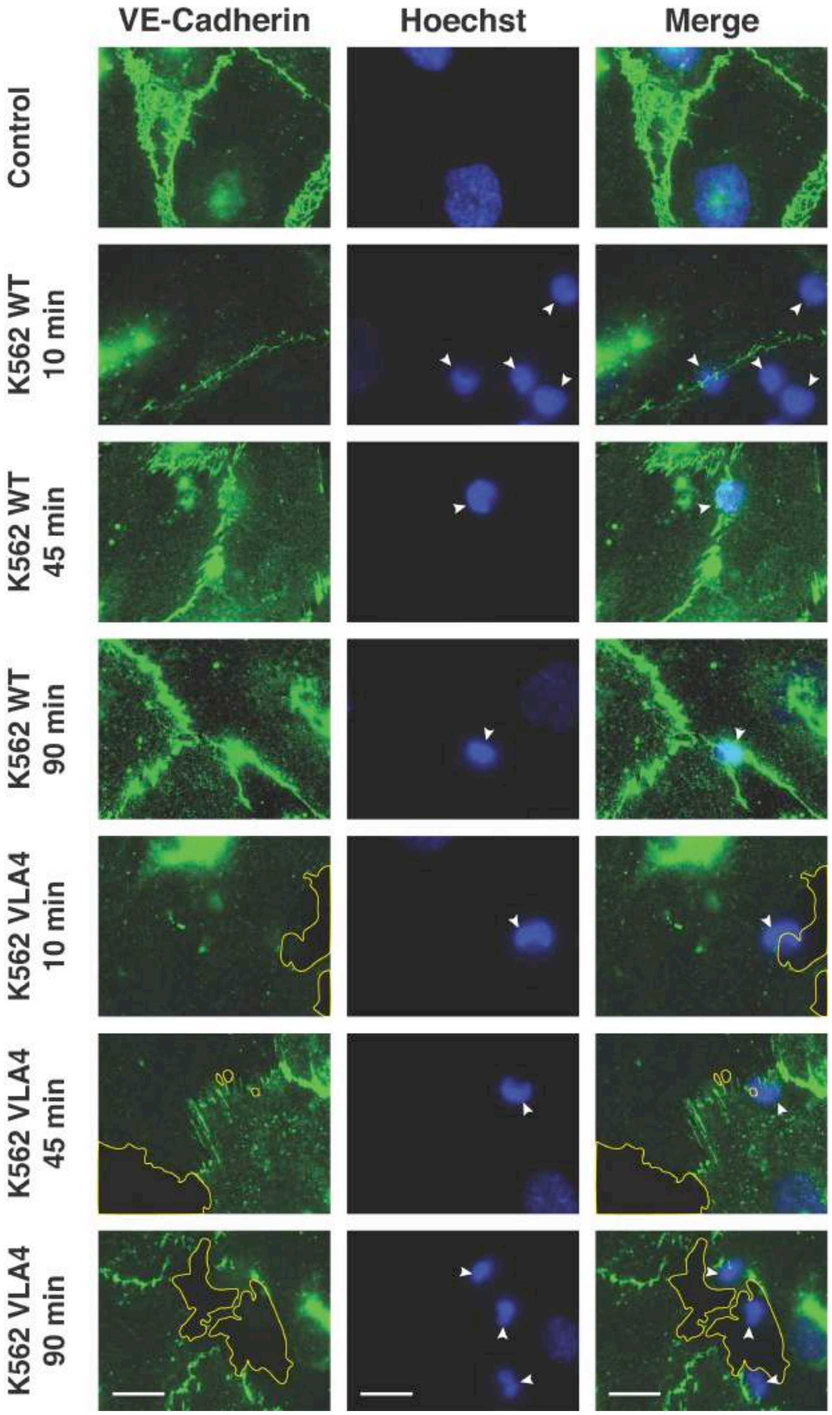

Supplemental Fig S3. (A) Representative images of HPMEC cells either left alone or treated with increasing concentrations of IL-8 for 45 min. (B) Representative images of HPMEC cells either left alone or treated with IL-8 at 20 ng/ml for 10, 45 and 90 minutes. (C) Representative images of HUVEC cells either left alone or treated with K562 WT or K562 VLA-4 cells for 10, 45 and 90 minutes. Gaps are outlined in yellow. Scale bars represent 10 μm.

Figure S4

A

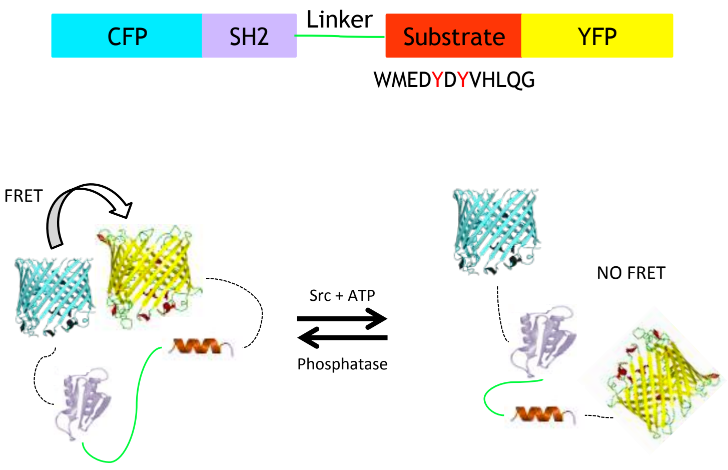

B

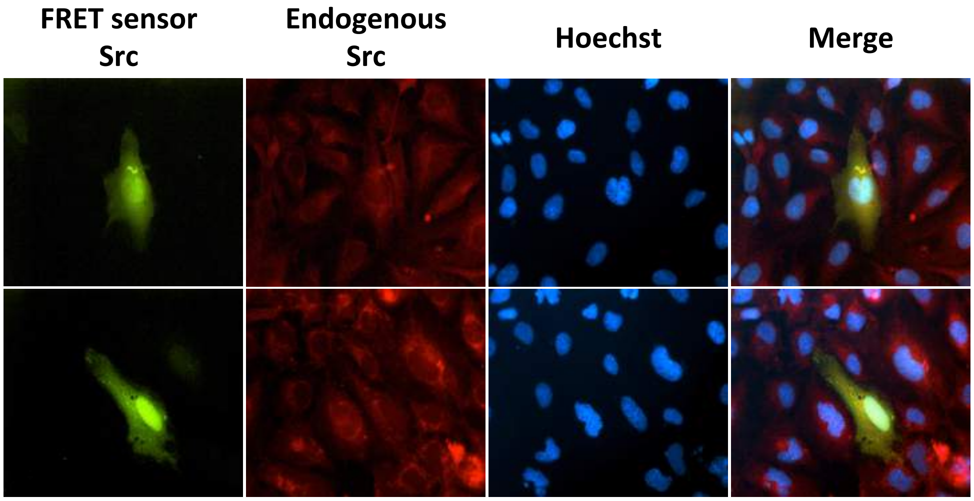

Supplemental Fig S4. (A) Src FRET biosensor design. (B) Src FRET sensor localizes throughout the cytoplasm. The staining pattern of HPMEC cells transfected with Src is similar to endogenous Src.

Figure S5

A

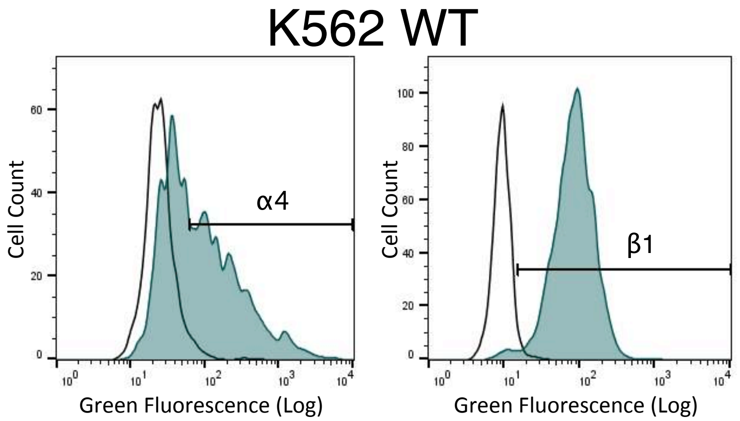

B

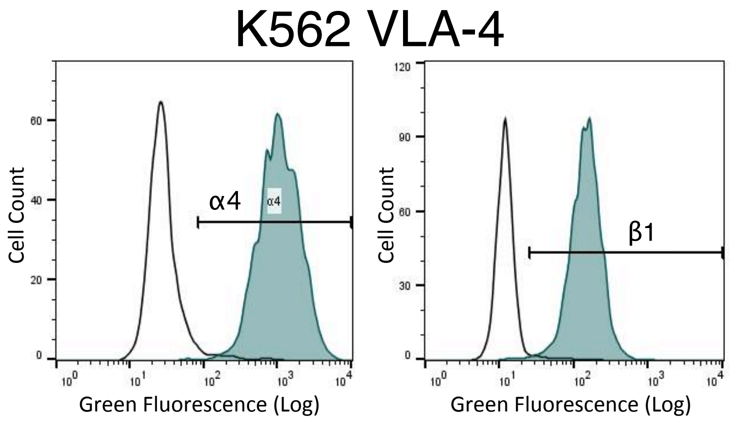

Supplemental Fig S5. Flow cytometry characterization of VLA-4 expression on cell surfaces. (A) VLA-4 receptor expression in K562-WT cells. (B) VLA-4 receptor expression in K562-VLA4 positive cells.
